# Reactive Oxygen Species and Antioxidants in Wound Healing: Mechanisms and Therapeutic Potential

**DOI:** 10.1111/iwj.70330

**Published:** 2025-04-27

**Authors:** Kelechi Ukaegbu, Edward Allen, Kathy K. H. Svoboda

**Affiliations:** ^1^ Department of Periodontology Texas A&M School of Dentistry Dallas Texas USA; ^2^ Private Practice Houston Texas USA; ^3^ Center for Advanced Dental Education Dallas Texas USA; ^4^ Department of Biomedical Sciences Texas A&M School of Dentistry Dallas Texas USA

**Keywords:** antioxidants, oxidative stress, reactive oxygen species, wound healing

## Abstract

Wound healing is a complex biological process encompassing haemostasis, inflammation, proliferation and matrix remodelling. Reactive oxygen species (ROS) play a pivotal role in regulating key events such as antimicrobial defence, platelet activation and angiogenesis. However, excessive ROS levels can induce oxidative stress (OS), disrupting the healing cascade and contributing to chronic wounds, inflammation and impaired tissue repair. Systemic conditions like diabetes, obesity, smoking and ageing further exacerbate OS, highlighting its clinical significance in wound management. Antioxidants (AOx), both endogenous and exogenous, have demonstrated therapeutic potential in mitigating OS, promoting wound closure and enhancing cellular recovery. Compounds like Vitamin E, curcumin, ferulic acid and resveratrol improve AOx enzyme activity, reduce oxidative damage and accelerate wound healing in multiple studies. Emerging evidence supports targeting oxidative pathways as a viable strategy to improve outcomes in chronic and systemic OS‐related conditions. This review explores the dual role of ROS in wound healing, the impact of OS in systemic diseases, and the therapeutic potential of AOx in fostering optimal healing outcomes, advocating for robust clinical trials to establish standardised interventions.

**Summary**

- Wound healing is a multifaceted and meticulously regulated process involving sequential phases: Haemostasis, inflammation, proliferation and matrix remodelling. Each phase is crucial for restoring tissue integrity and function following an injury.
- Reactive oxygen species (ROS) are integral to wound healing, contributing to antibacterial defence, platelet activation and angiogenesis. However, excessive ROS production can lead to oxidative stress, disrupting the balance between pro‐oxidants and antioxidants, which is associated with impaired wound healing, particularly in chronic wounds and metabolic conditions.
- The role of antioxidants in mitigating oxidative stress during wound healing is increasingly recognised. Antioxidants like Vitamins E and C, curcumin, ferulic acid and resveratrol have shown promise in promoting wound closure, reducing oxidative damage and enhancing the activity of antioxidant enzymes, presenting potential avenues for therapeutic intervention in improving wound healing outcomes.

Wound healing is a highly regulated, multifaceted biological process essential for tissue repair and regeneration. It progresses through distinct yet overlapping phases: hemostasis, inflammation, proliferation, and matrix remodeling. A delicate balance between cellular and molecular mechanisms orchestrates effective healing, while disruptions can lead to chronic wounds, fibrosis, or impaired tissue regeneration. Central to this process is the role of reactive oxygen species (ROS), which serve as crucial signaling molecules for immune activation, angiogenesis, and extracellular matrix remodeling. However, excessive ROS production can trigger oxidative stress (OS), exacerbating inflammation and delaying wound closure. Systemic conditions such as diabetes, obesity, smoking, and ageing further modulate this balance, often impairing the body's ability to resolve OS, thus prolonging inflammation and hindering tissue repair. Understanding the interplay between ROS, oxidative stress, and systemic diseases is critical in developing targeted therapeutic strategies to enhance wound healing outcomes.

## 1 Wound Healing

### 1.1 Haemostasis

Immediately after injury, damaged blood vessels rapidly contract, and a blood clot forms. Platelets, principal contributors to haemostasis and coagulation, become activated via platelet receptors that interact with extracellular matrix (ECM) proteins and promote adhesion to blood vessel walls. The clot primarily serves to plug the wound, prevent bleeding as well as provide protection against pathogen invasion. The clot also acts as a scaffold for incoming immune cells, harbouring a reservoir of cytokines and growth factors to guide wound cells in early repair; these include platelet‐derived growth factor (PDGF) and vascular endothelial growth factor (VEGF) [1].

### 1.2 Inflammation

The immune response is initiated by injury‐induced signals including damage‐associated molecular patterns (DAMPs) released by necrotic cells and damaged tissue, and pathogen‐associated molecular patterns (PAMPs) from bacterial components such as lipopolysaccharide [2]. A subsequent release of pro‐inflammatory cytokines namely interleukin 1 (IL‐1) and tumour necrosis factor‐alpha (TNF‐α) attracts circulating leucocytes to the site of injury [3]. This results in vasodilation, which, along with the expression of selectins, facilitates neutrophil and monocyte adhesion and diapedesis [1]. Neutrophils remove necrotic tissue and pathogens via phagocytosis and the release of ROS, antimicrobial peptides, and proteolytic enzymes [4]. They also trap and kill pathogens in an extruded web of DNA coated with antimicrobial peptides and cytotoxic histones, termed extracellular traps [5]. Inflammasomes, particularly NOD‐like receptor pyrin domain‐containing 3 (NLRP3) expressed in various immune and non‐immune cells, play a crucial role during the inflammatory phase of wound healing and are activated by DAMPs and PAMPs activate them. As a result, inflammatory cystine‐aspartic protease, caspase‐1 is activated, leading to the additional release of IL‐1β as well as pro‐inflammatory IL‐18, and IFN‐γ, the latter a key macrophage attractant [6]. In the absence of infection, neutrophils and inflammasomes decline within a few days of injury onset. Most neutrophils are extruded from the wound site as they adhere to the fibrin scab, whereas others are removed by macrophage efferocytosis [7].

Circulating monocytes enter the wound tissue where they differentiate into macrophages. Macrophages are master effector cells in tissue repair, displaying both versatility and high plasticity [8]. They reach peak wound infiltration 7 days post‐injury. Similar to neutrophils, macrophages are phagocytic and generate ROS; however, they exhibit differential behaviours and morphological changes in response to cytokines. Wound macrophages are traditionally separated into two main subsets: M1 mature inflammatory and M2 resolving anti‐inflammatory. M1 is a macrophage phenotype that prolifically produces pro‐inflammatory cytokines, such as TNF‐α and IL‐6 [9]. In the latter stages of inflammation, macrophages are characterised by a transition to M2, which occurs through neo‐differentiation of newly recruited monocytes or macrophages in situ switching. In this state, they express pro‐resolutory cytokines such as IL‐4, 10 and 13, which supports re‐epithelialization, fibroplasia and angiogenesis [10].

### 1.3 Proliferation

The proliferative phase of healing is characterised by extensive activation of keratinocytes, fibroblasts, macrophages and endothelial cells, which orchestrate wound closure, matrix deposition and angiogenesis respectively. As early as 12 h post‐injury, keratinocytes are activated by changes in mechanical tension, electrical gradients and cytokines [1]. Epidermal growth factor (EGF) binding to its receptor (EGFR) further stimulates keratinocyte proliferation and migration by promoting cytoskeletal reorganisation and enhancing cell motility, essential for effective wound closure [2]. They negotiate the debris and necrotic tissue of the wound bed through their interactions with the preliminary matrix, which contains matrix metalloproteinases (MMPs) and other proteases such as plasmin [11]. When keratinocytes from opposing edges meet, migration terminates and a thin epithelial layer is established.

Fibroblasts are the main cell type responsible for replacing the provisional fibrin‐rich matrix through degradation with substantial granulation tissue rich in fibronectin, collagen and proteoglycans. Resident and mesenchymal‐derived fibroblasts respond to signalling factors from platelets, endothelial cells and macrophages, including transforming growth factor beta (TGF‐β) and PDGF, directing fibroblasts to become either pro‐fibrotic, laying down ECM proteins, or differentiating into myofibroblasts which drive wound contraction [12].

New blood vessels are formed through angiogenesis to meet the metabolic demands of the highly proliferative healing tissue. This process is primarily triggered by hypoxia, and hypoxia‐inducible factor‐1α (HIF‐1α), leading to increased transcription of pro‐angiogenic genes such as VEGF [13]. VEGF stimulates microvascular endothelial cells to proliferate and migrate into the wound bed, where they sprout new vessels that fuse with others to form stable, tubular networks. In addition, PDGFs play a critical role in angiogenesis. PDGF‐AA primarily binds to PDGF receptor‐α (PDGFR‐ α) and supports the proliferation and migration of mesenchymal cells, aiding in tissue remodelling and vessel stabilisation. Conversely, PDGF‐BB binds both PDGFR‐α and PDGFR‐β, making it very potent in recruiting pericytes and vascular smooth muscle cells to newly formed vessels, thus enhancing vessel maturation and stability [14].

### 1.4 Matrix Remodelling

ECM remodelling spans the entire injury response, ending with the formation of a mature, type I collagen‐rich scar. Fibroblasts are vital in elastin formation; lysyl oxidase (LOX) facilitates efficient elastin deposition and stabilisation, which is crucial for restoring the mechanical (elasticity and flexibility) properties of tissues during healing, as well as collagen maturation. Myofibroblasts have an abundance of alpha‐smooth muscle actin, associated with an ability to generate strong contractile forces and close wounds [1]. There is evidence that myofibroblasts can revert to fibroblasts when exposed to growth factors, in particular fibroblast growth factors (FGF‐1 and 2) and heparin, emphasising that remodelling is a dynamic process [15]. The wound‐healing response stops when macrophages, endothelial cells and fibroblasts undergo apoptosis or exit the injury site. The resulting scar is functional, though it may not fully recapitulate the pre‐injury tissue architecture.

Oral and cutaneous wound healing progress through the same fundamental stages—haemostasis, inflammation, proliferation and remodelling, yet they differ markedly in their cellular and molecular dynamics. Cutaneous wounds are at higher risk of aberrant collagen deposition and impaired remodelling, which can result in hypertrophic scars and keloids. Oral wounds exhibit accelerated healing. Oral mucosal fibroblasts display a pro‐regenerative phenotype, characterised by higher proliferative capacity and reduced secretion of pro‐inflammatory cytokines compared to dermal fibroblasts [16]. Saliva also plays a pivotal role in promoting rapid oral wound healing, rich in antimicrobial agents and growth factors, including TGF‐β and lactoferrin, which not only accelerate tissue regeneration but also protect the wound site from microbial invasion. Moreover, the continuous moisture provided by saliva prevents scab formation, fostering an optimal environment for keratinocyte migration and promoting faster re‐ epithelialization [17].

## 2 The Role of Free Radicals in Wound Healing

Highly reactive free radical derivatives of oxygen (O_2_), known as ROS are paramount to this process acting as secondary messenger‐signalling molecules. ROS are produced by all cells during normal metabolic processes such as the electron transport chain in mitochondria. Particularly large amounts are generated in wounded and inflamed tissue through the ‘respiratory burst’ of phagocytes. In brief, pathogens are taken into phagolysosomes where enzyme complex NADPH oxidase converts O_2_ to a superoxide radical (•O_2_) which, under a dismutase reaction can be converted into hydrogen peroxide (H_2_O_2_) by enzyme superoxide dismutase (SOD). Myeloperoxidase (MPO), combines H_2_O_2_ with chloride to form hypochlorous acid (HClO) [18]. Additional ROS are generated via the Fenton reaction, in which ferrous iron (Fe^2+^) reacts with H_2_O_2_, generating highly reactive hydroxyl radicals (•OH) and hydroxide ions (OH−). Nitrogen can also create free radicalreactive nitrogen species (RNS). Nitric oxide (NO) is one of the most well‐known RNS produced by nitric oxide synthases (NOS), which convert L‐arginine into NO and citrulline. Inducible NOS (iNOS) is significantly involved in the immune response. Lastly, peroxynitrite (ONOO^−^) is another potent oxidant formed by the rapid reaction between NO and •O_2_.

### 2.1 The Role of Free Radicals in Promoting Wound Healing

ROS confers several advantages throughout the recovery process. Upon adhesion to exposed ECM, platelets produce ROS through NADPH oxidase, which causes degranulation of adenosine diphosphate and thrombin, recruiting and activating additional platelets to form the primary platelet plug [19]. Platelet activation can also take place through the formation of peroxynitrite [20]. ROS influence the coagulation cascade, upregulating the expression of tissue factor (TF) part of the extrinsic clotting system, on endothelial cells and monocytes [21], together with H_2_O_2_, which contributes to vasoconstriction through modulation of vascular smooth muscle excessive haemorrhage is prevented [22].

The most widely recognised role of ROS is in antimicrobial defence. HClO damages bacterial membranes, causing osmotic imbalance and targeting thiol groups in bacterial proteins, causing irreversible modifications that impair bacterial metabolism and virulence [23]. H_2_O_2_ demonstrates concentration‐dependent effects. At low concentrations (< 50 μM), H_2_O_2_ serves as a signalling molecule and activates chemotactic redox‐sensitive nuclear factor kappa B (NF‐κB), which enhances the expression of pro‐inflammatory cytokines and antimicrobial peptides [24]. At higher concentrations (> 100 μM), H_2_O_2_ exerts direct antimicrobial activity, generating hydroxyl radicals (•OH) This oxidative damage compromises bacterial viability and facilitates pathogen clearance from the wound environment [25]. NO also plays a crucial role in host defence and is primarily released by macrophages. NO exerts antimicrobial effects by diffusing into bacterial cells and interacting with intracellular components. It modifies bacterial proteins through S‐nitrosylation and inactivates enzymes involved in metabolic and replication pathways [26]. ROS are crucial regulators of angiogenesis; low concentration H_2_O_2_ induces the activation of several critical kinases, including the mitogen‐activated protein kinases (MAPKs) and phosphatidylinositol 3‐kinase (PI3K)/Akt pathway, which control endothelial cell proliferation, migration and tube formation—processes fundamental to angiogenesis [27]. ROS also stabilises HIF‐1α which is normally rapidly degraded. This mechanism ensures tissue hypoxia stimulates angiogenesis [28]. Finally, ROS stimulates the activation of collagen‐producing enzymes such as prolyl hydroxylase, essential for collagen maturation.

### 2.2 Antioxidants

**FIGURE 1:**
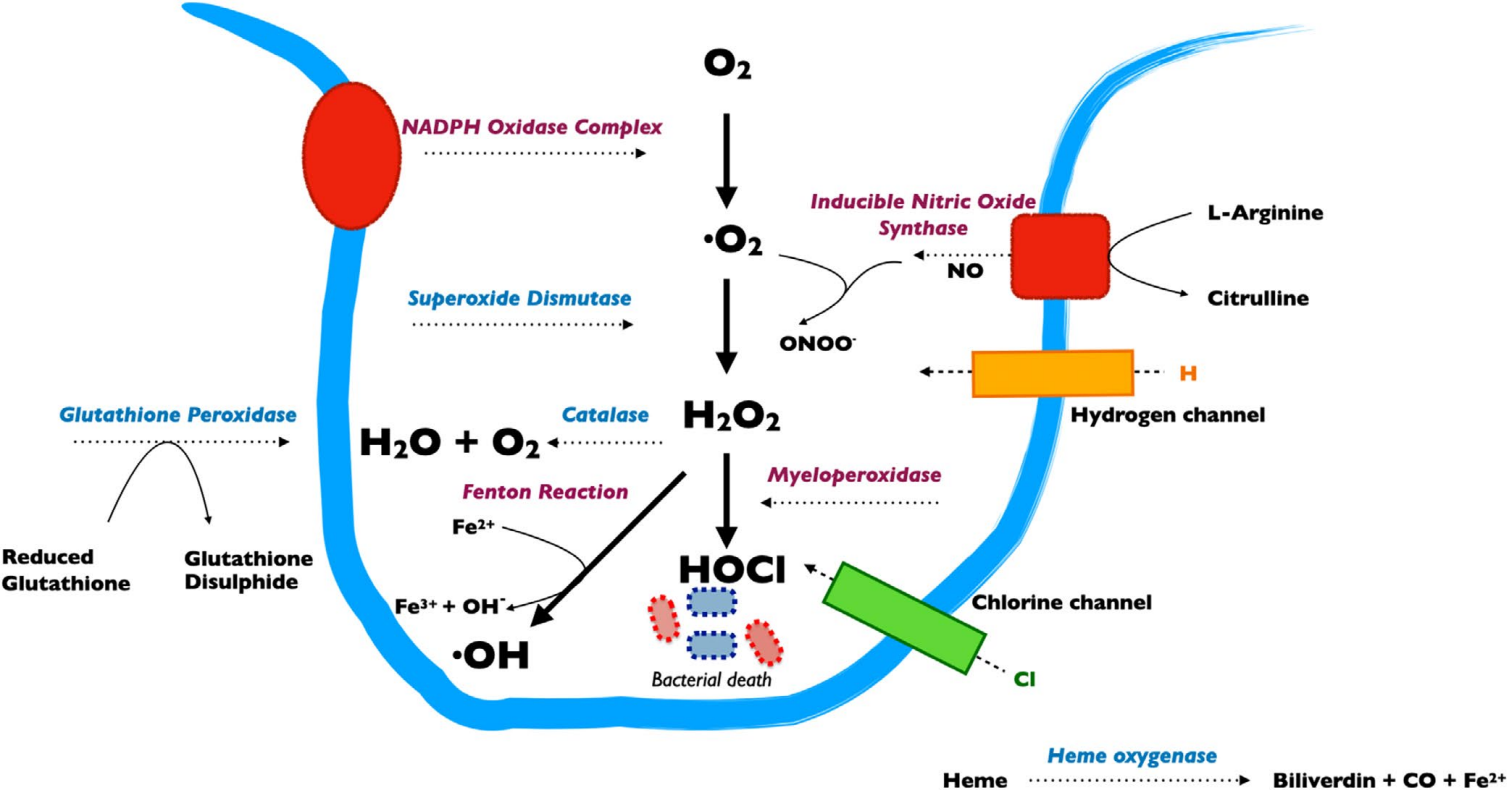
Summary of the generation of reactive oxygen and nitrogen species (ROS/RNS) in phagocytic cells, highlighting key enzymes such as NADPH oxidase, superoxide dismutase (SOD), and myeloperoxidase (MPO).

ROS lack pathogen specificity and through secretion into the extracellular milieu can cause ‘bystander damage’. Therefore, it is crucial to preserve the beneficial effects of ROS while minimising their potential harmful and degenerative impacts. Halliwell and Gutteridge succinctly defined an antioxidant (AOx) as “any substance that, when present at low concentrations compared with those of an oxidizable substrate, significantly delays or prevents oxidation of that substrate” [29]. AOx work in a variety of ways including direct ROS scavenging, inhibition of ROS precursors, and via upregulation of endogenous defences [30]. AOx can be first classified as endogenous or exogenous. Endogenous AOx can be enzymatic or non‐enzymatic. Enzymatic AOx include SOD which converts O_2_ into less potent H_2_O_2_. Catalase (CAT), which is expressed in high prevalence within peroxisomes decomposes H_2_O_2_ into H_2_O_2_ and O_2_. Glutathione peroxidase (GPx) also catalyses the reduction of H_2_O_2_ to H_2_O by oxidising reduced glutathione (GSH) to glutathione disulfide (GSSG) [31]. Inducible heme oxygenase (HO‐1), functions is a vital antioxidant enzyme by degrading pro‐oxidant heme into biliverdin, Fe^2+^ and carbon monoxide, biliverdin is subsequently converted to bilirubin. Non‐enzymatic AOx can be divided into metabolic or nutrient. Metabolic AOx are intrinsically produced, such as glutathione, bilirubin and lipoic acid. Whereas, nutrient AOx are obtained through diet or supplementation (e.g., vitamins‐based vitamin C and E, carotenoids, curcumin and ferulic acid). Lastly, master transcription factor nuclear factor erythroid 2‐related factor 2 (Nrf2) plays a crucial role in regulating endogenous AOx response through nuclear translocation and binding to the antioxidant response element (ARE) resulting in increased transcription of AOx enzymes [32]. The intricate interaction between endogenous ROS and AOx is summarised in Figure 1.

### 2.3 Oxidative Stress

Sies defined OS as a “disturbance in the pro‐oxidant‐antioxidant balance in favour of the former” [33] creating oxidation end products. Lipid peroxidation causes alterations in the structure and functionality of cell membranes, resulting in changes such as increased fluidity, heightened permeability, a decline in membrane potential, and eventual membrane rupture [34]. Products of lipid peroxidation, including malondialdehyde (MDA), 4‐hydroxyl‐2‐nonenal (HNE), and isoprostane, serve as crucial markers for evaluating both local and systemic oxidative damage. ROS can interact with DNA, a widely used biomarker is 8‐hydroxy‐deoxyguanosine (8‐OHdG) [35]. ROS induces modifications in protein functional activity which can be monitored via protein carbonyl (PC) groups [36]. As a final point, C‐reactive protein (CRP) is a widely used marker of systemic inflammation and can serve as an indirect indicator of OS. OS is a common factor in various systemic conditions, including obesity, diabetes and smoking‐all of which are associated with impaired wound healing. A key unifying biomarker across these conditions is high‐sensitivity C‐reactive protein (hs‐CRP), which reflects a state of low‐grade systemic inflammation. Elevated hs‐CRP levels have been consistently observed in individuals with the aforementioned conditions [37, 38], further underscoring its role in chronic inflammation and its connection to OS.

### 2.4 The Role of ROS in Chronic Wound Healing, Fibrosis and Scarring

Chronic wounds affect approximately 6.5 million people in the United States, imposing a financial burden of nearly $50 billion annually on the healthcare system [39]. These wounds are marked by inflammatory stagnation, minimal granulation tissue formation, and persistent activation of ROS‐generating leukocytes. Uncontrolled neutrophil activity in chronic wounds results in excessive ROS release, which activates NF‐κB which propagates inflammation, upregulation of IL‐1, 8, and TNF‐α resulting in a positive feedback loop. H_2_O_2_ induces endothelial cell expression of GMP‐140, enhancing leukocyte adhesion and sustained inflammation [40]. In addition, macrophages in chronic wounds are M1 dominant. Sindrilaru et al. reported that approximately 80% of macrophages at chronic wound margins are M1, releasing high levels of TNF‐α and •OH [41]. In vitro studies also show the phenotype switch is required on IL‐4 and 13 being present in the extracellular environment; if these cytokines are missing, M1 macrophages persist [42]. ROS activates the NLRP3 inflammasome in M1 macrophages, leading to the release of IL‐1β. This cytokine further amplifies inflammation, creating a feedback loop that further impairs M2 polarisation and stalls the healing process [43]. ROS disrupts angiogenesis as well as ECM formation, the former through inhibition of prolyl hydroxylase domain proteins, leading to the ‘over‐stabilisation’ of HIF‐1α even under normoxic conditions, resulting in vascular leakage that hinders tissue repair [44]. Oxidative damage impairs keratinocyte proliferation and migration, which are crucial for re‐epithelialisation. ROS‐mediated activation of NF‐κB and TNF‐α alters the secretion of MMPs, namely MMP‐1 and 3 from fibroblasts and MMP‐2 and 9 from macrophages; these unduly degrade the ECM [45].

The later stages of wound healing are prone to fibrotic and proliferative changes if OS is present by overactivating the differentiation of fibroblasts into myofibroblasts through the activation of TGF‐β. Myofibroblasts produce excessive amounts of collagen, fibronectin, and other ECM components, which lead to fibrosis. TGF‐β not only inhibits the apoptosis of myofibroblasts but can *suppress* MMPs, while simultaneously increasing the production of tissue inhibitors of metalloproteinases (TIMPs). This imbalance contributes to the uncontrolled deposition of ECM. Furthermore, OS activates the p38 MAPK pathway, which increases the activity of LOX, promoting abnormal collagen cross‐linking. These processes contribute to the development of keloids and hypertrophic scars [46].

## 3 The Impact of OS in Systemic Conditions and Wound Healing

OS is a common feature in several systemic diseases and conditions, including obesity, diabetes, smoking and ageing, all of which are associated with poor wound healing outcomes. Obesity is a chronic metabolic disease increasingly recognised as a significant risk factor for impaired wound healing, total wound failure and wound dehiscence [47, 48, 49]. This association is multifactorial, with OS and dysregulated adipokine secretion being pivotal contributors. Adipose tissue in obese individuals functions as an endocrine organ, releasing elevated levels of TNF‐α and IL‐6. These cytokines exacerbate systemic inflammation and amplify ROS production, creating an environment detrimental to cellular and tissue homeostasis [50]. Key adipokines, such as leptin and adiponectin, play crucial roles in modulating OS. Leptin, whose levels correlate directly with adipose tissue mass, is central to immune regulation and metabolism. However, obesity‐induced leptin resistance diminishes its effects and increases ROS levels. This resistance is associated with reduced activity of AOx enzymes (SOD, CAT and GPx). Conversely, adiponectin, an anti‐inflammatory and AOx adipokine, is markedly reduced in obesity, undermining its protective effects, such as inhibiting NF‐κB. Low adiponectin levels are also linked to impaired endothelial function and angiogenesis. The imbalance between pro‐inflammatory and anti‐inflammatory adipokines in obesity exacerbates OS and perpetuates a vicious cycle of inflammation and tissue injury [51].

Mitochondrial dysfunction is another critical feature of obesity that exacerbates OS. Nutrient excess, characterised by chronic overfeeding, leads to sustained activation of mitochondrial pathways to accommodate the surplus of energy substrates. This heightened activity overloads the electron transport chain, causing electron leakage and excessive ROS generation. Over time, mitochondrial damage accumulates, impairing their efficiency and releasing DAMPs into the cellular environment, which further stimulates inflammatory signalling, creating a self‐perpetuating loop of mitochondrial dysfunction and OS, ultimately impairing energy‐dependent wound repair processes such as cell migration and matrix deposition [52].

Endoplasmic reticulum (ER) stress emerges as another key player in the obesity –wound healing axis. In obese states, nutrient overload and lipid accumulation disrupt the ER's capacity to properly fold proteins, leading to the activation of the unfolded protein response (UPR). The UPR is initially protective, aiming to restore ER homeostasis by halting protein translation, increasing molecular chaperones and enhancing protein degradation pathways. However, chronic activation of the UPR results in pro‐inflammatory signalling through pathways such as c‐Jun N‐terminal kinase (JNK) and NF‐κB, along with the induction of apoptosis. This prolonged ER stress exacerbates systemic inflammation and compromises cellular functions essential for tissue repair [53]. Hyperglycemia, commonly associated with obesity and insulin resistance, exacerbates OS through multiple metabolic pathways including the activation of the polyol and hexosamine (maladaptive glucose metabolism) pathways, converging on protein kinase C (PKC) signalling, and augmented mitochondrial oxidative phosphorylation, all of which increase ROS production [54]. Dysfunctional adipocytes secrete excessive pro‐inflammatory mediators, skewing macrophage polarisation towards the M1 phenotype, which promotes chronic inflammation and tissue destruction. Encouragingly, bariatric surgery has been shown to restore immune balance and improve wound healing outcomes, highlighting the potential reversibility of some obesity‐related impairments [55].

Diabetes (DM) is a metabolic disorder characterised by chronic hyperglycemia due to insulin resistance, and/or impaired insulin secretion. The combination of microvascular complications (reduced blood flow and oxygen delivery) and macrovascular issues (impaired circulation) makes wound healing significantly slower in diabetic patients. Interestingly, wound complication rates vary by location, with facial wounds showing fewer complications than those on other parts of the body [56]. Like obesity, DM is associated with OS, but the mechanisms leading to OS in diabetes are more directly linked to disruptions in glucose metabolism and altered vascular function. The accumulation of advanced glycation end‐products (AGEs) is a hallmark of diabetic conditions and contributes to prolonged inflammation and impaired wound healing. AGEs form through the non‐enzymatic glycation of proteins and lipids in the presence of elevated glucose levels, and they interact with their receptor, RAGE, to activate inflammatory pathways [57]. Again, this interaction leads to skewed macrophage polarisation toward the M1 phenotype, which exacerbates chronic inflammation and delays the healing process [58]. Unlike obesity, where inflammatory cytokines are largely secreted by adipocytes, the AGE‐RAGE interaction in diabetes primarily activates NF‐κB and other pro‐inflammatory signalling pathways directly within endothelial and immune cells, further impairing wound healing [59]. Experimental studies have shown that hyperglycemia suppresses Nrf2 in diabetic macrophages, resulting in higher OS output. Conversely, activation of Nrf2 through chemical inducers significantly reduces OS and inflammation [60].

Tobacco smoking is a well‐established risk factor for poor wound healing, with numerous studies indicating prolonged healing times, reduced wound tensile strength and an increased risk of flap necrosis [61, 62, 63]. Smoking contributes to impaired tissue repair through several mechanisms, primarily driven by OS. One of the most significant effects of smoking is the upregulation of neutrophil activity, which induces a ‘hyperactive’ inflammatory response [64]. This results in excessive tissue destruction, delayed healing, and an overall pro‐inflammatory environment. In oral tissues, smoking has been shown to reduce the levels of SOD; both light and heavy smokers exhibit a dose‐related reduction in salivary and gingival crevicular fluid SOD levels compared to nonsmokers, suggesting that even minimal smoking exposure can impair the body's AOx defences [65]. Low levels of ROS exposure from tobacco smoke can also alter fibroblast morphology and microtubule organisation, impairing their mobility and function [66]. Additionally, another study found that human gingival fibroblasts (hGFs) exposed to nicotine for 17 h disrupt the translocation of beta1 integrin to the cell membrane, potentially compromising cell adhesion [67]. Combined with the inactivation of smoke‐induced ROS inactivation anti‐protease α1‐ antitrypsin [68] smokers are at increased risk of delayed healing and chronic fibrotic wounds.

Ageing is associated with a progressive decline in tissue integrity and regenerative capacity, which directly contributes to delayed wound healing. The accumulation of oxidative damage to cellular macromolecules results in cellular dysfunction, triggering a cascade that culminates in cellular senescence. Senescent cells no longer proliferate and accumulate in tissues, where they contribute to a persistent pro‐inflammatory environment—a phenomenon termed inflammaging. This state of chronic, low‐grade inflammation significantly impairs healing [69]. Senescent cells express a senescence‐associated secretory phenotype (SASP) which is characterised by the secretion of pro‐inflammatory cytokines, ROS and MMPs, which degrade the ECM and hinder the formation of a stable tissue scaffold. The presence of ROS in ageing tissues leads to a vicious cycle, where oxidative damage induces cellular dysfunction, which, in turn, increases ROS production and delays the transition from the inflammatory to the proliferative phase of wound healing [70]. Moreover, impaired angiogenesis is affected. Age‐related changes in the vascular endothelium, coupled with increased OS, impair VEGF signalling, resulting in inadequate tissue perfusion and oxygenation [71].

## 4 OS as a Therapeutic Target

To address the detrimental effects of OS in both oral and cutaneous wounds, targeting ROS as a therapeutic approach has been explored and found to be advantageous in various studies (see Table 1). Most research on the AOx properties of vitamins has predominantly concentrated on vitamin E, either on its own or in combination with other vitamins and supplements. Vitamin E is an AOx that neutralises free radicals by donating a hydrogen atom; its lipid‐soluble nature protects cell membranes from lipid peroxidation. The effects of palm vitamin E (PVE) and α‐ tocopherol (α‐Toc) (vitamin E derivative) supplementation on wound healing in diabetic rats have been investigated. Compared to unsupplemented rodents, both AOx groups demonstrated rapid wound closure with increased total protein content suggestive of enhanced cell proliferation. This correlated with histological signs of well‐organised healing tissue. SOD and GPx were significantly higher while MDA reduced, highlighting AOx capacity. Overall, PVE conferred superior benefits compared to α‐Toc [72]. In another study dietary AOx supplementation with vitamin C and E (VCE) and the combination of vitamin C, E and N‐acetylcysteine (NAC) (Comb) significantly modulated OS and accelerated wound closure rates in diabetic mice compared to controls, with noticeable improvements beginning as early as day 2 post‐wounding. Both VCE and Comb groups exhibited significantly lower blood glucose levels relative to the control DM rodents. Although glucose levels did not fully normalise, the reduction was positively correlated with faster wound closure, highlighting the link between glycaemic control and wound healing efficiency. Regarding AOx enzymes, at 48 h post wounding, HO‐1 in all groups was raised. However, at 72 h, HO‐1 levels in VCE and Comb were barely detectable. Furthermore, SOD levels in the VCE and Comb groups decreased at 48 h compared to the DM group. The authors proposed that dietary AOx may have a beneficial effect on shortening the inflammatory response by regulating ROS production during the inflammatory phase, thereby accelerating overall wound healing [73]. Barbosa et al. examined the effects of combined AOx supplementation with vitamins C, E and zinc on OS and wound healing in paediatric burn injuries. The AOx‐supplemented group demonstrated a markedly accelerated wound healing rate compared to the control group. OS was notably reduced in the AOx group, as evidenced by a significant decrease in MDA levels. Plasma vitamin E levels significantly increased in the supplemented group, confirming absorption and utilisation of the supplemented vitamin E. However, serum levels of vitamin C and zinc did not exhibit significant changes, although the AOx group showed a trend towards increased zinc levels. The rapid consumption of vitamin C can be attributed to its hydrophilic nature, while zinc, as a trace element, is naturally present in smaller quantities [74].

**TABLE 1:** Summary of antioxidant‐based interventions and their effects on wound healing, antioxidant enzymes, and inflammatory markers across different models, dosages, and delivery methods.

Antioxidant	Dosage	Delivery	Model	Wound type	Outcome
Palm vitamin E alpha tocopherol [72]	Palm vitamin E (PVE) 200 mg/kg α‐Tocopherol (α‐Toc) 200 mg/kg	Oral	Streptozotocin‐induced diabetic rodents	Full‐thickness excision wound	**PVE > α‐Toc** ↑ Wound closure rate ↑ Epithelization and collagen ↑ Total protein content ↑ GPx & SOD ↓ MDA
VCE (Vitamins C & E) Comb (Vitamin C, E & N‐acetyl cysteine) [73]	Vitamin C 0.5% & vitamin E 0.5% Vitamin C 0.5%, vitamin E 0.5% & N‐acetyl cysteine 2.5%	Oral	Alloxan monohydrate induced diabetic rodents	Full‐thickness excision wound	**VCE = Comb** ↑ Wound closure rate ↑ Glycemic control ↑ Liver vitamin E ↓ iNOS ↓ COX‐2
Vitamin E, C & zinc [74]	Vitamin E 1.5× upper intake level Vitamin C 1.35× upper intake level Zinc 2× recommended daily dietary allowance	Topical	Paediatric burn patients	Burn injury	↑ Plasma vitamin E ↓ Healing time ↓ MDA
Curcumin [75]	Curcumin 40 mg/kg	Topical	Rodent	Full‐thickness excision wound	↑ Wound closure rate ↑ Wound tensile strength ↑ Epithelization rate ↑ ECM ↑ Collagen & collagen crosslinking ↑ SOD, CAT & GPs ↓ MDA
Curcumin [76]	Curcumin 5 mg	Topical	Rodent	Full‐thickness excision wound	↑ Wound closure rate ↑ Collagen & collagen crosslinking ↑ Caspase‐3 ↑ SOD & GSH ↑ Nrf2 ↓ ROS ↓ MDA
Curcumin‐loaded chitosan alginate‐ cyclodextrin (CA‐CD‐Cur) sponge [77]	Curcumin 4.17 mg/sponge	Topical	Rodent	Full‐thickness excision wound	↑ Wound closure rate ↑ Vascularized granulation tissue ↑ Collagen and collagen crosslinking ↑ TGF‐β1 ↓ NF‐ĸB ↓ SOD ↓ MDA
Ferulic acid [78]	Ferulic acid (Oral 1) 10 mg/kg/daily Ferulic acid (Oral 2) 20 mg/kg/daily Ferulic acid (Topical) 2% daily	Oral and topical	Streptozotocin‐induced diabetic rodents	Full‐thickness excision wound	Oral 2 > Oral 1 > Topical ↑ Wound closure rate ↑ Granulation tissue ↑ Epithelization ↑ NO ↑ Glycemic control ↑ SOD, GSH & CAT ↓ MDA
Resveratrol [79]	Resveratrol 1 μM	Direct exposure	Swiss albino fibroblasts (In vitro)	Oxidative stress model (H_2_O_2_)	↑ Cell proliferation ↑ COL1A2 mRNA ↓ ROS
Resveratrol [80]	Resveratrol 10 μM	Intradermal injection	Streptozotocin‐induced diabetic rodents	Full‐thickness excision wound	↑ Wound closure rate ↑ Collagen ↑ Myofibroblast markers ↑ M2 macrophage markers ↓ IL‐1β, IL‐6 & TNF‐α
Resveratrol combinations [81]	Resveratrol, ferulic acid, tetrahydrocurcuminoids (RFT) all at 10^−5^ M Phloretin, ferulic acid & resveratrol (PFR) all at 10^−5^ M Phloretin, ferulic acid & tetrahydrocurcuminoids (PFT) all at 10^−5^ M	Direct exposure	Human gingival fibroblasts (hGFs) and periodontal ligament fibroblasts (hPDLFs) (In vitro)	Oxidative stress model (H_2_O_2_, ethanol & nicotine)	↑ Cell viability ↓ ROS
Resveratrol combinations [82]	**Single agent** Ferulic acid (F), phloretin (P), resveratrol (R), tetrahydrocurcuminoid (T) all at 10^−5^ M **Double Combination** Resveratrol & ferulic acid (RF) resveratrol & phloretin (RP) resveratrol & tetrahydrocurcuminoid (RT) Phloretin & ferulic acid (PF) phloretin & tetrahydrocurcuminoid (PT) ferulic acid & tetrahydrocurcuminoid (FT) all at 10^−5^ M **Triple Combination** Resveratrol, ferulic acid & tetrahydrocurcuminoids (RFT) Phloretin, ferulic acid & resveratrol (PFR) Phloretin, ferulic acid & tetrahydrocurcuminoids (PFT) all at 10^−5^ M	Direct exposure	Human gingival fibroblasts (hGFs) and periodontal ligament fibroblasts (hPDLFs) (In vitro)	Oxidative stress model (nicotine) & scratch‐wound assay	↑ Cell viability ↑ Cell motility ↑ RacGTP
Phloretin and ferulic acid [83]	Patented composition (Proprietary dose)	Topical	Rodent	Gingival augmentation	↑ Healing scores ↑ TNF‐α ↑ SOD
Platelet‐rich plasma and mesenchymal stem cells [84]	Platelet‐rich plasma (PRP) 4.8 × 10^6^ platelets/wound Mesenchymal stem cells (MSCs) 1 × 10^5^ cells/wound Combined (PRP + MSCs) 4.8 × 10^6^ platelets/wound +1 × 10^5^ cells/wound	Intradermal injection	Rodent	Full‐thickness burn wound	**Combined > MSC > PRP** ↑ Wound closure rate ↑ Epithelization ↑ VEGF ↑ SOD, CAT & GSH ↑ Bcl‐2 ↓ TNF‐α, MMP‐9 &TGF‐β1 ↓ Capase‐3 & Bax ↓ NF‐ĸB ↓ MDA
Buffy coat, platelet‐rich fibrin & platelet‐poor plasma lysates [85]	Buffy coat (BC) 10% (v/v) Platelet‐rich fibrin (PRF) 10% (v/v) Platelet‐poor plasma (PRP) 10% (v/v)	Direct exposure	Human gingival fibroblasts (hGFs) (In vitro)	Oxidative stress model (H_2_O_2_)	**BC > PRF > PPP** Intrinsic CAT activity ↓ Necrosis

Curcumin is a potent polyphenolic antioxidant, which is present in Indian curry spice turmeric with antimicrobial, anti‐inflammatory and antioxidant properties. Its chemical structu re, particularly the phenolic and diketone groups, allows the donation of an electron or hydrogen atom to stabilise free radicals. Panchatcharam et al. reported wounds treated with topical curcumin contracted and re‐epithelialized significantly faster along with increased tensile strength compared to controls. Histopathological analysis confirmed enhanced collagen synthesis and maturation throughout all healing phases evidenced by a 3‐fold increase in acid‐soluble collagen content. MDA levels were reduced by 50%, indicating decreased OS. Additionally, AOx enzymes namely, SOD, CAT and GPX were significantly elevated, collectively enhancing free radical scavenging and reducing oxidative damage [75]. A recent study, randomly divided rodents into control and curcumin groups following full‐thickness wound induction. The wound healing rates of the curcumin group were higher than those of the control group with complete healing by day 14 whereas control wounds persisted and resulted in greater scar tissue. AOx‐supplemented mice demonstrated higher collagen fibre synthesis which was well organised in a ‘basket weave pattern’ resembling healthy dermal tissue. Furthermore, the levels of ROS and MDA in the curcumin group were lower than the controls, conversely, the level of Nrf2, SOD and GSH were higher than in the control group. Interestingly, an increase in executioner caspase‐3 was reported, the authors theorise that this phenomenon reflects apoptosis of excess inflammatory cells and fibroblasts reducing the risk of scarring [76]. Although curcumin‐loaded chitosan alginate‐cyclodextrin (CA‐CD‐Cur) sponges have been shown to improve wound healing morphologically, they were associated with reduced NF‐ĸB and SOD levels, the latter finding contrasts with most of the existing literature on curcumin's effects. One possible interpretation is that curcumin's direct radical‐ scavenging activity reduced the levels of •O2 minimising the need for SOD activity. However, when •O2 is neutralised, and more H_2_O_2_ is formed as a by‐product, therefore greater reliance on CAT [77].

Ferulic acid (FA) is a phenolic compound containing a —OH group and conjugated double carbon bond enabling hydrogen atom or election donation. It is a natural AOx found in many staple foods, such as fruits, vegetables, cereals and coffee. The effect of (topical, high and low dose) FA was investigated in diabetic rats resulting in FA‐treated rats having a significant decrease in wound area when compared with controls. In addition, FA applied on a wound for 14 days significantly increased SOD, GSH and CAT, whereas MDA concurrently lowered. High oral dose FA was most effective at improving healing parameters and glycemic control [78].

Reversterol (RSV) isolated from the roots of *Veratrum grandiflorum* is a natural polyphenol compound with potent levels of AOx owing to the presence of the —OH groups on the aromatic rings, with the ability to donate electrons. RSV application to fibroblasts significantly reduced OS stress levels in response to H_2_O_2_ incubation and improved proliferation and migration [79]. Recent in vivo studies have also found that RSV is capable of M2 macrophage polarisation, reducing the release of pro‐inflammatory cytokines [80]. RSV, in combination with other agents, has been proven to reduce OS in oral fibroblasts in vitro after exposure to stressors such as H_2_O_2_, nicotine and ethanol. Cell viability of hGFs and periodontal ligament (hPDLF) fibroblasts recovered following exposure to AOx. A combination of phloretin, ferulic acid and tetrahydrocurcuminoid (PFT) exhibited the highest recovery in hPDLF, whereas the phloretin, ferulic acid and resveratrol (PFR) formulation was highly effective in enhancing the viability of both HGF and HPDLF. PFR demonstrated the most pronounced reduction in ROS levels, particularly in cells subjected to ethanol and nicotine exposure, highlighting potent AOx capacity [81]. The authors previously investigated the effects of nicotine on oral wound healing by examining its impact on hGF and hPDLFs. Their findings demonstrated that nicotine impairs wound healing by increasing ROS and inhibiting cell migration by reducing RacGTP levels. However, treatment with AOx combinations (particularly double and triple) effectively restored the latter, enhancing cell motility and viability. Among all the tested treatments, PFR and RFT demonstrated the most potent effects [82].

In a recent animal study, the application of an AOx gel containing phloretin and ferulic acid significantly enhanced clinical wound healing compared to bisbiguanide chlorhexidine treatment and control. The AO‐treated group exhibited superior healing scores, with notable improvements in wound colour, reduced swelling and minimal graft exposure. Gene expression analysis further supported these findings; TNFα was elevated in the AO group at 24 h, suggesting a prompt and controlled early inflammatory response essential for effective healing. Additionally, SOD was significantly higher in the AO group at the same time point, reflecting enhanced AOx defence mechanisms that likely contributed to reduced oxidative damage [83].

Lastly, autologous products have demonstrated AOx properties alongside improvements in wound healing. Tamman et al. investigated the therapeutic effects of bone marrow‐derived mesenchymal stem cells (MSCs) and platelet‐rich plasma (PRP) on burns in rats. Rodents were divided into MSCs, PRP, combined MSCs and PRP and control groups. Combined therapy significantly accelerated wound contraction and shortened the epithelialization period compared to other groups. Enhanced angiogenesis was observed, indicated by increased VEGF, while TGF‐β was reduced, the latter improving scar regulation. Treatment also alleviated inflammation by decreasing pro‐inflammatory markers (TNF‐α, IL‐6, NF‐κB, NO and iNOS) expression. However, in contrast to Wu and Deng [76], apoptosis was mitigated, as evidenced by decreased levels of Caspase‐3 and Bax, alongside increased expression of the anti‐apoptotic marker Bcl‐2 [84].

An in vitro study examining the effects of platelet‐rich fibrin (PRF) and its components on H_2_O_2‐_induced OS in human gingival fibroblasts. The researchers found that PRF, platelet‐poor plasma (PPP), and buffy coat lysates could neutralise H_2_O_2‐_induced toxicity and prevent necrotic cell death. The underlying mechanism was attributed to CAT; when CAT activity was blocked using inhibitors or heat treatment, the protective effects were abolished. Notably, the study ruled out apoptosis as a cause of cell death by showing that caspase‐3, a marker of apoptosis, was not activated during H_2_O_2_ exposure. Additionally, the use of conditioned media from PRF membranes showed protective effects when harvested after 24 h but not after 72 h, indicating a time‐dependent activity [85].

Although the existing studies demonstrate positive outcomes, there is substantial variability in experimental design, including differences in wound models, AOx formulations, dosages, delivery methods and outcome measures. This lack of standardisation not only complicates the comparison of results across studies but also limits the robustness of conclusions regarding the efficacy of AOx interventions in wound healing. Furthermore, inconsistencies in the interpretation of findings further hinder the establishment of a cohesive understanding of their mechanisms of action. Notably, the majority of these studies have been conducted in rodent models, which, while valuable, may not fully recapitulate human pathophysiology. Therefore, there is a pressing need for well‐designed, mechanistic investigations and rigorously conducted human randomised controlled trials to elucidate the therapeutic potential of AOx in modulating OS and enhancing wound healing in clinical practice.

**FIGURE 2:**
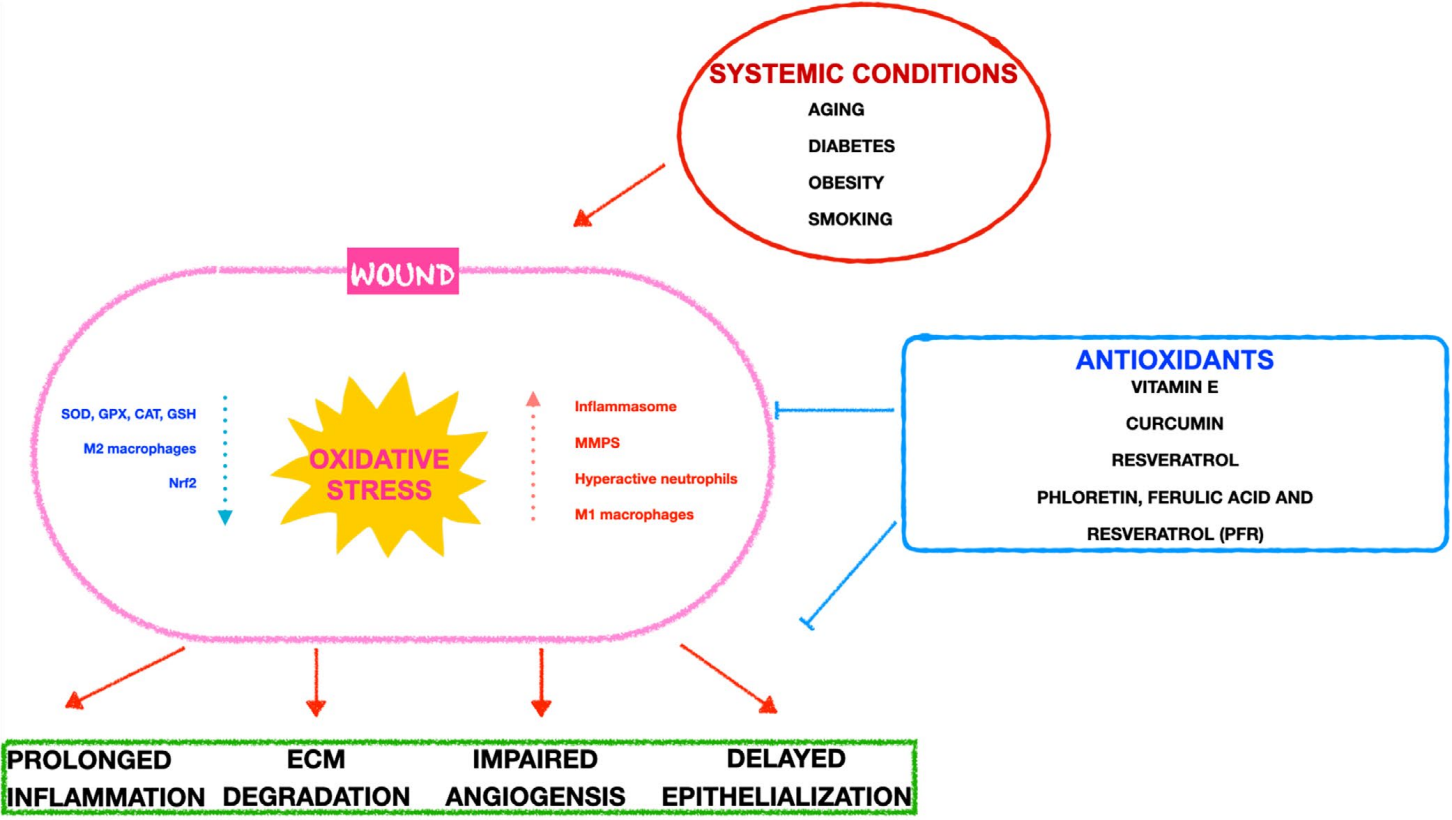
Summary of the role of oxidative stress in delayed wound healing and prolonged inflammation, and the confounding effect of systemic diseases like diabetes and obesity. Antioxidants such as vitamin E and resveratrol can help mitigate damage.

OS significantly influences the trajectory of wound healing. Although ROS plays a crucial role in the early stages of healing by eliminating pathogens and promoting cellular activities, an excessive and sustained presence can derail the process. The impact of OS extends beyond wounds, affecting broader systemic health. Conditions like obesity and diabetes, marked by chronic inflammation and oxidative imbalance, show how these systemic factors can impede the healing cascade. Recognising OS as a therapeutic target opens avenues for intervention. AOx, ranging from vitamin E to autologous products, demonstrates promise in mitigating ROS‐induced damage and fostering a conducive environment for healing (summarised in Figure 2).

## Conflicts of Interest

The authors declare no conflicts of interest.

## Data Availability

Data available on request from the authors.
